# Mechanism Analysis of Amphotericin B Controlling Postharvest Gray Mold in Table Grapes

**DOI:** 10.3390/foods14071260

**Published:** 2025-04-03

**Authors:** Yingying Wu, Jingyi Wang, Shenli Wang, Yijie Ke, Tianyi Ren, Ying Wang

**Affiliations:** College of Ocean Food and Biological Engineering, Jimei University, Xiamen 361021, China

**Keywords:** postharvest disease, antifungal agent, *Botrytis cinerea*, antifungal mechanism, transcriptomics

## Abstract

Gray mold, caused by *Botrytis cinerea*, is the primary factor contributing to postharvest losses in table grape fruit. In this study, we have identified amphotericin B (AMB), a natural compound originating from *Streptomyces nodosus*, as a promising agent in managing postharvest gray mold in table grapes. In vitro experiments demonstrated that 0.2 mg/L AMB achieved an inhibition rate of over 90% against *B. cinerea* in PDA medium, and in vivo assays on grape berries showed that 200 mg/L AMB treatment could completely suppress the occurrence of gray mold disease. A mechanism analysis found that AMB treatment disrupted the plasma membrane structure, which consequently triggered cellular leakage and induced cell death. Furthermore, AMB application effectively modulated the transcriptional profile of genes related to redox homeostasis, transmembrane transport, and peroxidase functions in *B. cinerea*, thereby reducing the virulence of the fungus. In addition, AMB treatment had the potential to activate defense mechanisms in table grapes by enhancing the activities of ROS-scavenging enzymes and defense-associated enzymes. Collectively, AMB can be regarded as a natural antifungal agent that effectively combats *B. cinerea*, thereby extending the postharvest shelf life of table grape fruit.

## 1. Introduction

Grape (*Vitis vinifera* L.), a highly perishable non-climacteric fruit, has been a major component of human diets for thousands of years across the globe. Unfortunately, their sensitivity to postharvest decay, primarily triggered by *Botrytis cinerea*, significantly limits their storage duration [1,2]. *B. cinerea* frequently inflicts enormous economic losses as it is capable of infecting all above-ground portions of grapevines, encompassing both ripe and unripe berries. Moreover, the spread of this disease is expedited when infected berries come into direct contact with nearby healthy berries during transportation and storage processes [3,4,5]. Over recent decades, the primary interventions for mitigating postharvest losses have remained low-temperature storage and synthetic fungicides application. However, the effectiveness of cold storage in controlling gray mold is limited, as *B. cinerea* can grow and sporulate even at temperatures down to **−0.5 °C** [4]. Relying solely on low-temperature preservation fails to adequately control the occurrence of gray mold [4,6]. Consequently, chemical control continues to serve as the dominant strategy for managing *B. cinerea*. Nonetheless, intensive fungicide applications pose significant environmental contamination risks and human health hazards [7,8]. As a result, the pursuit of low-toxicity and eco-friendly natural antimicrobials has become exceedingly urgent.

In recent years, microbial-derived agricultural antibiotics have garnered considerable attention. Notably, dactylimicin, a vital constituent of agricultural antibiotics, enjoys widespread application in China, successfully mitigating crop decay and protecting around 13 million acres of agricultural land each year [9,10]. Furthermore, salinomycin, known for its strong ability to suppress the growth of pathogenic bacteria, has become a widely adopted agent in agricultural practices [11]. Up until now, numerous studies have documented the application of diverse microbial secondary metabolites as active components for preventing and managing postharvest diseases that are triggered by an array of phytopathogenic fungi, such as tanespimycin [12], natamycin [13,14], lucensomycin [4], and rapamycin [12,15]. Amphotericin B (AMB), a medically crucial antifungal agent, is naturally produced by *Streptomyces nodosus* [16]. Furthermore, AMB has received approval from the Food and Drug Administration (FDA) as an effective broad-spectrum antifungal medication [17,18], with its safety for human use having been rigorously confirmed [19]. A recent study has shown that high-dose AMB liposome successfully treated *Candida albicans* infection within the amniotic sac of a 25-week pregnant woman, allowing the pregnancy to continue and result in the delivery of a healthy newborn [20]. Similarly, intravitreal injection of AMB has been shown to be a safe and effective treatment modality for *Candida glabrata*-induced endophthalmitis [21]. Moreover, *Aspergillus fumigatus* stands out as one of the prevalent pathogenic fungi, posing a significant threat to immunocompromised patients with high incidences of morbidity and mortality [22]. AMB remains the cornerstone drug in the treatment of *A. fumigatus* infections [22]. In addition, an investigation has been conducted on the inhibitory capabilities of AMB in controlling citrus fruit decay induced by *Penicillium italicum*, unveiling its distinctive anti-plant–pathogenic–fungus attributes [17]. However, the potential efficacy in controlling gray mold in grapes and the underlying inhibitory mechanism of AMB remain unknown.

Accordingly, this study was designed to evaluate the efficacy of AMB in inhibiting *B. cinerea* both in vitro and in vivo, thereby addressing a gap in the current literature. Additionally, the underlying mechanisms of action were elucidated through a combination of transcriptomic analysis, fluorescence microscopy, and physiological and biochemical assays. Collectively, these findings provide a comprehensive technical validation and theoretical foundation for the potential commercialization of AMB as a novel and highly effective antifungal agent.

## 2. Materials and Methods

### 2.1. Reagent

AMB compound (750 mg/g, CAS. NO. 1397-89-3) was procured from Yuanye Biotechnology, located in Shanghai, China.

### 2.2. Pathogen Culture

The *B. cinerea* (B05.10) was generously supplied by Prof. Paul Tudzynski (Institut für Biologie und Biotechnologie der Pflanzen, Münster, Germany) [23,24] and cultivated on a potato dextrose agar (PDA) medium under white light at 22 °C. After a ten-day incubation period, the conidia were collected and suspended in potato dextrose broth (PDB), where the suspension was adjusted to a concentration of 2 × 10^5^ conidia/mL [14].

### 2.3. Fruit

Mature, mechanically undamaged, and uniformly sized table grapes (*Vitis vinifera* L. cv. Victoria) were harvested from a vineyard in Xiamen, China, in July 2024 and promptly transported to the laboratory on the same day.

### 2.4. In Vivo Antifungal Efficacy of AMB Towards B. cinerea

Table grapes were disinfected in a 2% sodium hypochlorite (Xilong Scientific, Shanghai, China) solution for 2 min, rinsed with clean water, and air-dried. Each grape was then punctured at the equator with a sterile pin to create a 2 mm wide and 6 mm deep wound. A 5 μL conidia suspension (2 × 10^5^ conidia/mL) was inoculated into each wound. After drying, the wounds were treated with 10 μL of AMB at concentrations of 0, 25, 50, 75, 100, and 200 mg/L. The grapes were incubated at 22 °C and 95% humidity for 3 days (d). Lesion diameters were measured every 24 h. Each treatment was replicated three times, and each replicate had 20 fruit.

### 2.5. In Vitro Antifungal Efficacy of AMB Towards B. cinerea

AMB was added into the PDA medium (90 mm diameter) in varying final concentrations, specifically 0, 0.05, 0.1, 0.15, 0.2, and 0.4 mg/L. Following 10 d of cultivation, the conidia were collected, and the conidia suspension was diluted to a density of 2 × 10^5^ conidia/mL. Subsequently, 5 μL of this conidia suspension was carefully applied to the center of the culture medium, which were then incubated at 22 °C. The diameters of the colonies were accurately measured at intervals of 24, 48, and 72 h. For the spore germination assay, a spore suspension of *B. cinerea*, with a concentration of 1 × 10^6^ conidia/mL, was cultured in a PDB medium containing a range of AMB concentrations (0, 0.2, 0.4, 0.8, and 1.0 mg/L) and incubated at 22 °C to detect both germination rates and the lengths of germ tubes. Gemination was confirmed when the length of the germ tube surpassed the diameter of the conidium. The germination rate is ascertained by computing the ratio of germinated spores to the total spores examined. The lengths of the germ tubes were meticulously measured using an eyepiece micrometer, following the procedures outlined in He et al. (2019) [14]. A random sample of approximately 200 spores was examined in each field of view, with the entire experimental procedure carried out on three repetitions to guarantee precision.

### 2.6. Determination of Cell Viability

Based on our previous study [25], fluorescein diacetate (FDA) was used to determine cell viability. Spores of *B. cinerea* were placed in the PDB medium (1 × 10^6^ conidia/mL) containing 0, 0.5, and 1.0 mg/L of AMB and then incubated with shaking (200 rpm) at 22 °C for 2 h. Afterward, the conidia were harvested by centrifugation at 8000× *g* for 5 min and subsequently assessed for cell viability using 50 mg/L FDA. Immediately after staining, the conidia were examined under a fluorescence microscope (Leica DM5000B, Wetzlar, Germany). This experiment was conducted in triplicate to ensure accuracy and reliability.

### 2.7. Determination of Malondialdehyde (MDA) Content, Electrical Conductivity, and Cellular Leakage

After culturing the conidia in the PDB medium for 3 d, AMB was supplemented into the growing mycelium at final concentrations of 0, 0.5, and 1.0 mg/L. Afterward, the cultures were then incubated for varying durations, specifically 0, 2, 4, 6, and 8 h. The levels of MDA were used to evaluate the extent of membrane lipid peroxidation, with the measurement conducted according to the procedures detailed by Cui et al. (2021) [26].

A spore suspension was inoculated into 100 mL of PDB to achieve a final spore concentration of 2 × 10^5^ conidia/mL. Following 3 d of shaking cultivation (200 rpm) at 22 °C, the mycelium was harvested and thoroughly rinsed with sterile distilled water. Afterward, 3 g of the mycelium were resuspended in 25 mL of sterile distilled water containing AMB at concentrations of 0, 0.5, and 1.0 mg/L. The mycelia were then cultured at 22 °C, 200 rpm for durations of 0, 2, 4, 6, and 8 h, during which electrical conductivity measurements were taken utilizing a conductivity meter (EC215, HANNA Instrument, Shanghai, China). To mitigate any potential interference from the background conductivity of AMB, the outcomes are expressed as relative variations from the initial reading.

The measurement of cell leakage in *B. cinerea* treated with AMB was conducted following a previously described method with slight modifications [27]. A 50 mL spore suspension was incubated at 22 °C and 200 rpm for 3 d and then harvested. Following this, the mycelium was resuspended in 30 mL of sterile water containing 0, 0.5, and 1.0 mg/L of AMB, respectively. The cultures were further incubated in a shaking incubator at 22 °C for 2, 4, 6, and 8 h. After removing the mycelium, the filtrate was assessed for the leakage of nucleic acids and soluble carbohydrates. The leakage of soluble carbohydrates was quantitatively determined using an anthrone reagent, while the leakage of nucleic acids was quantified by measuring the absorbance at 260 nm. For the quantification of protein leakage, we followed the method outlined by Liu et al. (2020) [28].

### 2.8. RNA-Sequencing

The spores of *B. cinerea* were first cultivated in 100 mL of PDB at 22 °C with shaking (200 rpm) for 3 d, followed by the addition of AMB to a final concentration of 1.0 mg/L. As a control, a separate set of mycelia was treated with sterile distilled water. After being cultivated for 2 h under identical conditions, the mycelium is separated by centrifuging at 8000× *g* for a duration of 10 min. This collected mycelium was then rinsed twice with PBS buffer, gently dried using filter paper, and promptly frozen in liquid nitrogen. Subsequently, total RNA was isolated from both sets of mycelia employing Trizol reagent (Tiangen, Beijing, China). The RNA samples obtained were then processed for cDNA library construction and transcriptome analysis according to the comprehensive protocol detailed by Zhang et al. (2022) [29].

### 2.9. RT-qPCR Analysis

The extraction of total RNA was carried out as mentioned above. For cDNA synthesis, the Reverse Transcription Premix Kit with gDNA Eraser (Accurate Biology, Changsha, China) was utilized, with a total RNA input of 1.0 μg. The gene-specific primers were designed utilizing the Primer3Plus online platform. Available online: https://www.primer3plus.com/ (accessed on 24 April 2024). The detailed information of the primers is provided in Table S1. RT-qPCR was performed on a StepOne Plus Real-Time PCR system from Applied Biosystems, utilizing SYBR Premix Ex Taq reagent sourced from Vazyme Biotech (Nangjing, China). The RT-qPCR procedure comprised an initial step of heating to 95 °C for 10 min, followed by 40 cycles with a two-step thermal profile of 95 °C for 15 s and 60 °C for 30 s. Gene transcription levels are then quantitated and compared using the comparative cycle threshold (Ct) 2 ^(−ΔΔCt)^ method.

### 2.10. Assays of Enzyme Activity

The determination of antioxidant enzymes activities, encompassing superoxide dismutase (SOD), catalase (CAT), and peroxidase (POD), was conducted in adherence to previous studies by Zhang et al. (2023) [30]. Briefly, grape berry powder (1 g) was mixed with 3 mL of phosphate buffer (pH 7.0, 50 mM), followed by centrifugation at 10,000× *g* for 20 min at 4 °C. The clear supernatant obtained was utilized for the enzyme activity assay. Additionally, the measurements of defense-related enzyme activities, namely phenylalanine ammonia-lyase (PAL), chitinase (CHI), and β-1,3-glucanase (GLU), were assayed using the procedures described by Li et al. (2022) [31]. The procedure for preparing the supernatant is described as follows: Grape samples (2 g) were homogenized in a boric acid/borax buffer (4 mL, pH 7.4) containing 3% PVPP, 0.037 g of NaCl, and 0.137 mL of β-mercaptoethanol per 100 mL at 0 °C. The homogenate was then centrifuged at 10,000× *g* for 20 min at 4 °C to obtain the supernatant, then determined the enzyme activity of PAL. For the analysis of CHI and GLU enzyme activities, a 0.5 g portion of the grape sample was mixed with 5 mL of disodium hydrogen phosphate–citric acid buffer (50 mmol/L, pH 5.0), homogenized at 0 °C, and then centrifuged at 10,000× *g* for 20 min at 4 °C. The resulting supernatant was collected for the determination of CHI and GLU activities.

### 2.11. Statistical Analysis

The data were statistically evaluated with the aid of SPSS software version 21.0 (IBM, Chicago, IL, USA). The results obtained from repeated trials in each experiment were expressed as mean ± standard deviations. The significance of the data was assessed using Duncan’s multiple range test or Student’s *t*-test, with a *p*-value < 0.05 considered statistically significant. Duncan’s multiple range test was employed for multiple comparisons, as it is well-suited for detecting significant differences among multiple treatment groups. The sample size was determined based on preliminary experiments and relevant references [1,17,27] to ensure the reliability and validity of the results.

## 3. Results

### 3.1. AMB Demonstrates Efficient Management of Gray Mold in Table Grape Fruit

**Figure 1:**
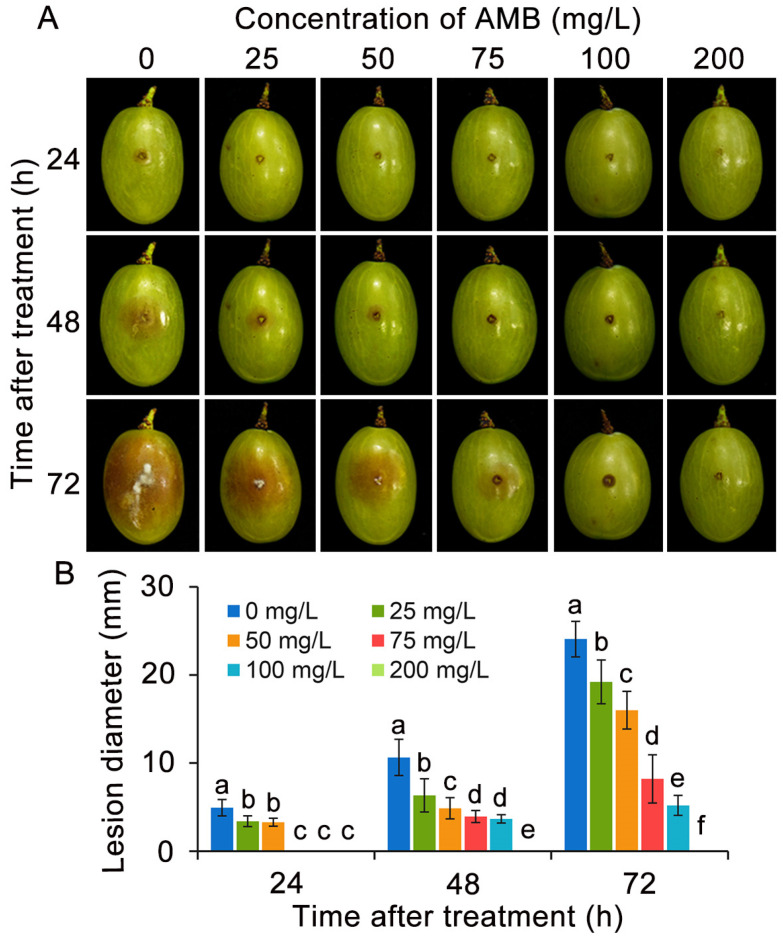
Treatment with 0.5 mg/L AMB reduced the lesion diameter of gray mold on table grapes by 54%. (**A**) Alterations in the morphology of table grapes during various storage periods across diverse treatment groups. (**B**) Lesion diameter. The significant differences (ANOVA, *p* < 0.05) among the treatment groups, as indicated by lowercase letters, were determined through Duncan’s multiple range test.

To evaluate the in vivo effectiveness of AMB in combating *B. cinerea* infections, comprehensive experiments were carried out on table grape fruit. As shown in Figure 1, following 48 h treatment with 25 mg/L AMB, the lesion diameter of table grapes was significantly reduced by approximately 40% compared to the control group, whereas with 50 mg/L AMB, the lesion diameter decreased by 54%. After 72 h of storage, the application of AMB at a concentration of 50 mg/L on table grapes resulted in a lesion diameter that was 65% of the untreated control, while grapes treated with 75 mg/L of AMB exhibited a lesion diameter that was just 34% of the control group. Furthermore, grapes administered with 100 mg/L of AMB remained disease-free after 24 h, and upon reaching 72 h, the diameter of lesions observed was just 21% of the untreated control. Remarkably, the application of 200 mg/L of AMB achieved absolute suppression in the occurrence of gray mold disease among table grapes during the whole experiment period.

### 3.2. AMB Impacts the Mycelial Growth and Spore Germination of B. cinerea

**Figure 2:**
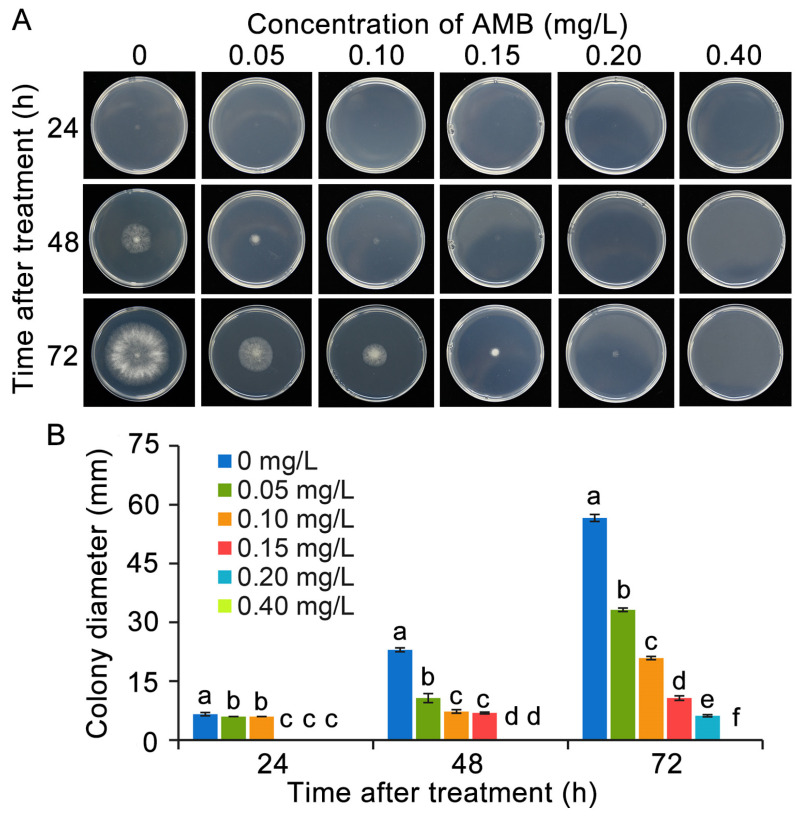
Inhibition of *B. cinerea* mycelial growth by AMB treatment. (**A**) The mycelia morphologies of *B. cinerea*. (**B**) Colony diameter. The significant differences (ANOVA, *p* < 0.05) among the treatment groups, as indicated by lowercase letters, were determined through Duncan’s multiple range test.

As the cultivation time progressed, the diameter of *B. cinerea* colonies in the control group steadily expanded. However, the growth of *B. cinerea* was effectively inhibited by AMB in a dose-dependent manner (Figure 2). Specifically, at a concentration of 0.05 mg/L, AMB notably reduced the colony diameter to less than half of the untreated control. After 72 h of cultivation, the colony diameters of the groups exposed to 0.15 mg/L and 0.20 mg/L of AMB decreased by 80% and 90%, respectively, in comparison to the control group. Moreover, 0.40 mg/L of AMB was able to completely suppress the mycelia growth of *B. cinerea* on PDA medium.

**Figure 3:**
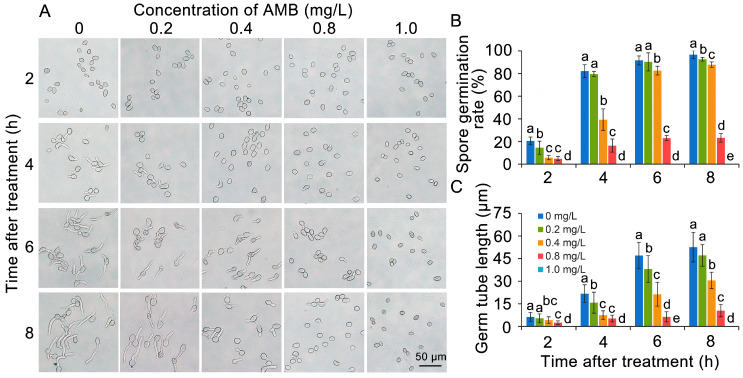
AMB exhibits an effective inhibitory effect on spore germination in *B. cinerea*. (**A**) Alterations in the morphology of *B. cinerea* spores in different treatment groups. (**B**) Spore germination rate. (**C**) Germ tube length. Bar = 50 μm. The significant differences (ANOVA, *p* < 0.05) among the treatment groups, as indicated by lowercase letters, were determined through Duncan’s multiple range test.

The inhibitory potential of AMB towards spore germination and germ tube elongation intensifies obviously with rising concentration levels. As shown in Figure 3, when exposed to a concentration of 0.4 mg/L AMB, the spore germination rate was observed to be 5.8% within 2 h and increased to 39.3% after 4 h. However, it was notably lower than that of the untreated control. In comparison, the untreated control spores attained a germination rate of 96.6% in 8 h, whereas the spores exposed to 0.4 mg/L AMB achieved just 87.8%. Furthermore, the germination rate of the 0.8 mg/L treatment group drastically dropped to 23.3%, with the germ tube length being just 10.4 μm, measuring only 20% of that in the untreated control group. In addition, the application of 1.0 mg/L AMB resulted in the complete inhibition of spore germination in *B. cinerea*.

### 3.3. AMB Treatment Impaired Cell Viability of B. cinerea

**Figure 4:**
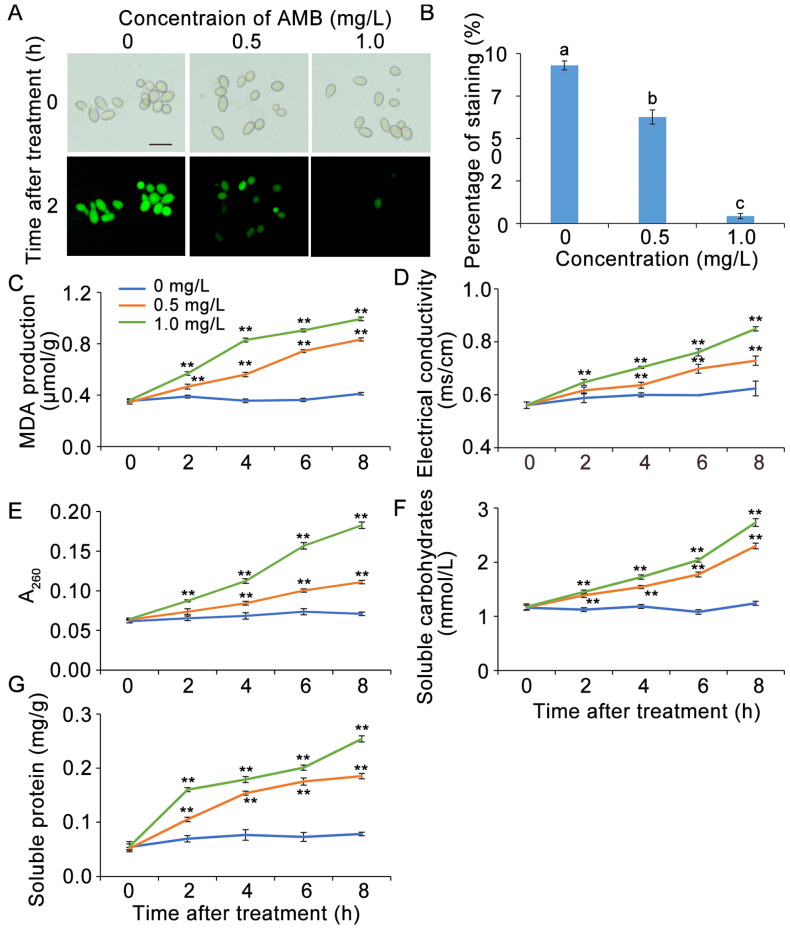
The impact of AMB on cell viability and membrane integrity in *B. cinerea*. Spores that lost cell viability exhibited green fluorescence (**A**), and a subsequent analysis was conducted to determine the proportion of spores affected (**B**). Bar = 20 μm. The extent of lipid peroxidation in the cell membrane is denoted by the MDA content (**C**), whereas the permeability of the membrane is expressed through electrical conductivity measurements (**D**). AMB-triggered cellular leakage was shown, including nucleic acids (**E**), soluble carbohydrates (**F**), and soluble proteins (**G**). The significant differences (*p* < 0.05) among the treatment groups, as indicated by asterisks, were determined through Student’s *t*-test.

The FDA staining was employed to assess the viability of spores following AMB treatment. The presence of green fluorescence in the cells indicates their robust vitality. As depicted in Figure 4A,B, nearly all spores in the control group underwent FDA staining, whereas spores treated with 0.5 mg/L AMB displayed a remarkably reduced FDA staining percentage and significant attenuation in fluorescence intensity. At a concentration of 1.0 mg/L, AMB virtually eliminated spore staining, suggesting a dose-dependent reduction in the cell vitality of *B. cinerea*.

### 3.4. AMB Treatment Influenced the Generation of MDA, Electrical Conductivity, and Cellular Leakage in B. cinerea

To assess membrane lipid peroxidation in *B. cinerea* treated with AMB, MDA content was measured. The MDA levels in AMB-treated groups increased with the cultivation time and AMB concentration, showing a positive correlation. In contrast, control group MDA levels remained stable (Figure 4C). After 8 h, MDA levels in 0.5 and 1.0 mg/L AMB-treated groups rose significantly by 2.4 and 2.8 folds, respectively, compared to the control.

Membrane lipid peroxidation compromises membrane integrity and increases cellular leakage. Electrical conductivity is a key indicator of membrane permeability. We examined the impact of AMB on the electrical conductivity of *B. cinerea*. Results showed that conductivity in AMB-treated groups increased with treatment time and AMB concentration, while the control group remained stable (Figure 4D).

The cellular leakages of *B. cinerea* were then assessed. As expected, leakage of nucleic acids, soluble carbohydrates, and soluble proteins increased in a dose-dependent manner with AMB concentration. After 8 h, the nucleic acid leakage was 1.7 times higher in the 0.5 mg/L AMB group and 2.8 times higher in the 1.0 mg/L group compared to the control (Figure 4E). Soluble carbohydrate content was 2.3 mmol/L and 2.8 mmol/L in the 0.5 mg/L and 1.0 mg/L AMB groups, respectively, versus 1.2 mmol/L in the control (Figure 4F). Soluble protein leakage also increased significantly with AMB concentration (Figure 4G). These results indicated that AMB treatment triggered membrane lipid peroxidation, disrupted membrane permeability, induced cellular leakage, and ultimately led to cell death.

### 3.5. RNA-Seq Analysis of B. cinerea After Treatment with AMB

**Figure 5:**
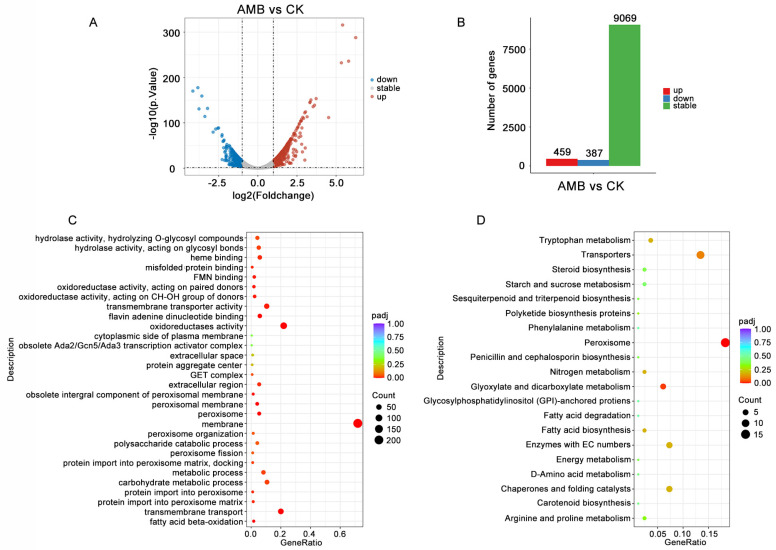
Transcriptome analysis comparing the AMB treatment group with the control group. (**A**) The volcano plot displays a visualization of the variance in differentially expressed genes (DEGs). Specifically, red dots highlight genes that are significantly up-regulated, blue dots depict genes that are significantly down-regulated, and gray dots signify genes that remain relatively unchanged in their expression levels. (**B**) DEGs between control and AMB-treated group. (**C**) GO annotations analysis of DEGs. (**D**) KEGG enrichment analysis of DEGs.

To delve deeper into how AMB inhibits *B. cinerea*, a transcriptome analysis was performed. On average, each sample yielded 78.6 million total clean reads, of which around 98.79% were effectively aligned to the reference genome of *B. cinerea* for the analysis of gene expression. A distinct set of 846 differentially expressed genes (DEGs) were shown when the AMB-treated group was compared to the control group. Of these, 459 genes underwent upregulation, whereas 387 genes exhibited downregulation (Figure 5A,B).

Gene Ontology (GO) enrichment analysis was conducted on AMB-treated and control samples. As illustrated in Figure 5C and Table S2, a significant proportion of the DEGs were localized in the membrane, encompassing an enrichment of 212 genes. Based on the GO functional annotation, the up-regulated DEGs predominantly exhibited involvement in oxidoreductase activity, flavin adenine dinucleotide binding, and fatty acid beta-oxidation. On the other hand, the down-regulated DEGs primarily contributed to oxidoreductase activity, transmembrane transport functions, and carbohydrate metabolic processes. The KEGG pathway analysis indicated that a significant proportion of the up-regulated DEGs were implicated in peroxisomal function, glyoxylate, and dicarboxylate metabolism, as well as chaperone activity and protein-folding catalysis, while the DEGs displaying a downregulation in expression were associated with transporter mechanism, tryptophan metabolism, and steroid biosynthesis (Figure 5D and Table S2).

### 3.6. Confirmation of Gene Expression via RT-qPCR

**Figure 6:**
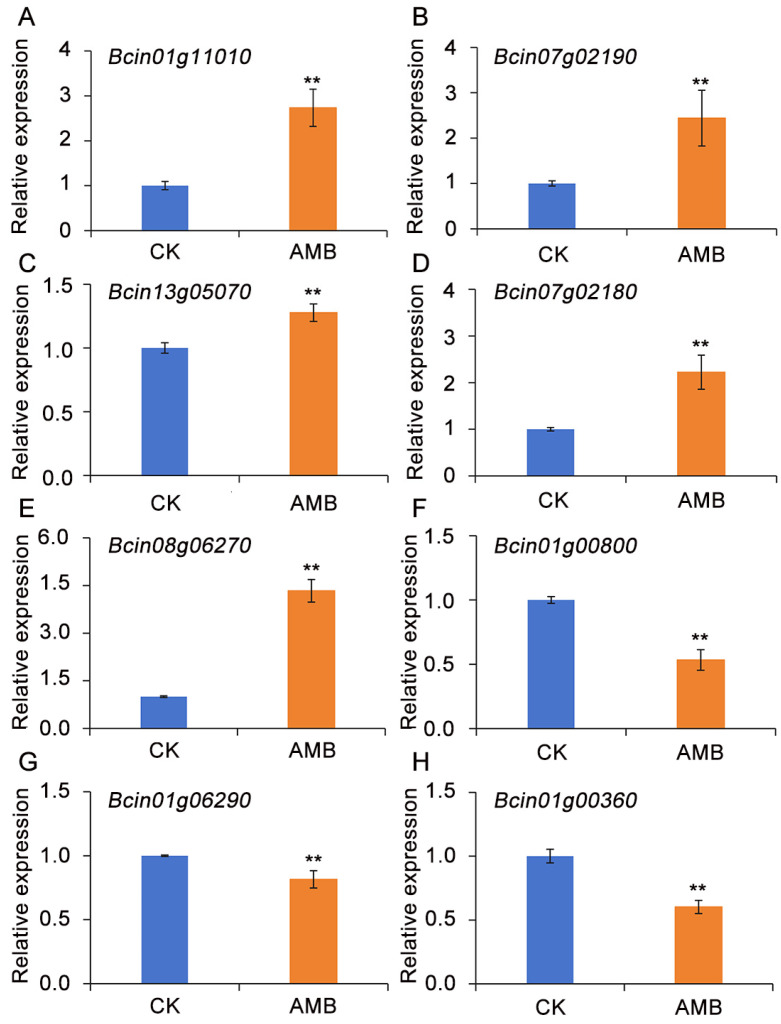
Verification of the relative expression levels of eight randomly selected DEGs from the transcriptome data by RT-qPCR. (**A**–**H**) represent the expression of *Bcin01g11010*, *Bcin07g02190*, *Bcin13g05070*, *Bcin07g02180*, *Bcin08g06270*, *Bcin01g00800*, *Bcin01g06290*, and *Bcin01g00360*, respectively. The significant differences (*p* < 0.05) among the treatment groups, as indicated by asterisks, were determined through Student’s *t*-test.

The expression profiles of a subset of DEGs identified via RNA sequencing have been validated through RT-qPCR. Our targeted analysis of eight DEGs, randomly selected from distinct KEGG pathways, revealed a strong alignment with the original RNA-Seq data, thus firmly validating the accuracy and reliability of our sequencing outcomes (Figure 6).

### 3.7. AMB Treatment Induced the Resistance Response of Table Grapes Against B. cinerea

**Figure 7:**
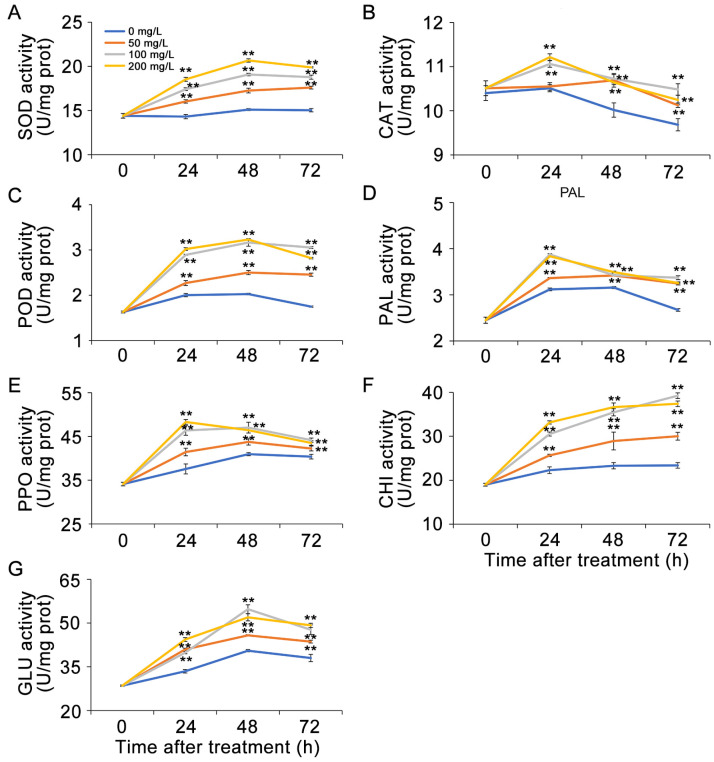
Impact of AMB treatment on the enzymatic activities of SOD (**A**), CAT (**B**), POD (**C**), PAL (**D**), PPO (**E**), CHI (**F**), and GLU (**G**) in table grapes infected with *B. cinerea*. The significant differences (*p* < 0.05) among the treatment groups, as indicated by asterisks, were determined through Student’s *t*-test.

This study examined the effects of *B. cinerea* infection on antioxidant and defense-related enzymes in table grapes. Results showed that AMB-treated grapes had significantly higher activities of SOD, CAT, and POD during storage compared to the control group. SOD and POD activities peaked at 48 h post-treatment, while the CAT activity was highest at 24 h (Figure 7A–C). The results indicated that AMB effectively activated the antioxidant enzyme system in table grapes.

AMB treatment also markedly enhanced the activity of defense-related proteins in table grapes. After 24 h of storage, PAL and PPO activities were highest, with 1.23- and 1.29-fold increases, respectively, in the 200 mg/L AMB group compared to the control (Figure 7D,E). GLU activity initially rose within 48 h then declined but remained above control levels (Figure 7F). CHI activity consistently increased throughout storage and correlated positively with AMB concentration (Figure 7G). These findings demonstrated that AMB coordinately activated multiple defense-related proteins to enhance grape disease resistance.

## 4. Discussion

*B. cinerea* is a crucial pathogen that annually results in substantial yield losses in grape cultivation globally [32]. Additionally, the oxidation triggered by *B. cinerea* negatively impacts not only the quality of grapes but also the taste and character of the resulting grape musts and wines [33]. Natural products are widely recognized for their environmentally friendly nature and minimal toxicity, making them a preferred choice for managing postharvest fungal decay [34]. AMB, originating from *S. nodosus*, is extensively utilized in the field of human health, particularly for treating systemic fungal infections [35]. While its therapeutic efficacy and safety profile in medical applications are well-established, potential food safety implications of AMB require further investigation through comprehensive in vivo studies and regulatory assessments. Currently, the application of AMB in combating postharvest fungal diseases remains under-explored. Up to now, it has only been specifically reported in the management of citrus rot caused by *P. italicum* [17]. Here, we found that AMB exhibits a remarkable inhibitory effect on the development of gray mold on table grapes, indicating that *B. cinerea* is highly susceptible to AMB. Notably, the highest AMB concentration applied in this study was 200 mg/L, with a maximum actual dosage of 1.5 μg per treatment (based on 10 μL application volume and 750 mg/g AMB concentration), which is deemed to be a safe level [20,36].

The pathogenesis of gray mold starts with spores firmly attaching to the plant surface, followed by germination and the production of germ tubes [1]. Subsequently, these germ tubes penetrate the protective skin of the fruit and colonize the plant tissues by forming intricate, highly differentiated hyphal networks, ultimately leading to the development of grape gray mold [1]. In the present study, AMB displayed significant suppression in hyphal development, spore germination, as well as germ tube elongation, implying that its efficacy in combating grape gray mold is likely related to its direct suppression of hyphal growth and spore maturation. The underlying mechanism of this phenomenon may be associated with the damage caused by AMB to the cell membrane, as well as the subsequent changes in the intracellular environment. These combined effects likely impede the normal extension of the germ tube. Prompted by these findings, we proceeded to examine the cellular viability, lipid peroxidation, and membrane permeability of *B. cinerea* under AMB treatment. The FDA-staining results showed that AMB significantly reduced the viability of *B. cinerea* spores. The integrity of the cell membrane is essential for maintaining cellular stability and function. Many antifungal agents work by disrupting the cell membrane structure [25,26]. MDA content and electrical conductivity are key indicators of membrane lipid peroxidation and membrane permeability, respectively [37]. Our study found that AMB increased MDA levels and electrical conductivity, suggesting its potential to attenuate the pathogenicity of *B. cinerea* by damaging the cell membrane. Further experiments confirmed this, showing AMB caused the release of cellular contents such as nucleic acids, soluble carbohydrates, and proteins from *B. cinerea* cells. Moreover, GO analysis of transcriptome data revealed significant AMB-induced alterations in membrane-related gene expression, particularly in fatty acid metabolism, β-oxidation, and sterol biosynthesis pathways. These findings strongly support our hypothesis that AMB primarily targets *B. cinerea* plasma membrane integrity and function.

The peroxisome, a single-membrane organelle found in nearly all eukaryotic cells, serves as the site for numerous vital metabolic reactions, including fatty acid β-oxidation and reactive oxygen species (ROS) degradation [38,39]. According to previous studies, peroxisomes are integral to the growth, development, and pathogenicity of various plant pathogenic fungi, including *Magnaporthe oryzae* [40], *Fusarium graminearum* [41], *Colletotrichum lagenarium* [42], and *B. cinerea* [39]. The research conducted by Li et al. (2022) [31] has verified that *BcPex8*, *BcPex10*, and *BcPex12* are indispensable for the growth and virulence of *B. cinerea*. However, our transcriptome analysis indicated that following AMB treatment, the expression of nine *Bcpex* genes, including *Bcpex8*, was remarkably up-regulated, aligning with the expression pattern of genes linked to fatty acid β-oxidation. The results imply that altered peroxisomal activity through *Bcpex* overexpression could represent another mechanism regulating *B. cinerea* virulence. Furthermore, peroxisome-mediated ROS scavenging is essential for fungal growth and pathogenicity [43,44]. As critical signaling molecules, ROS regulate cellular redox status and downstream pathways [45]. Many antifungal agents exert their effects by inducing ROS accumulation, causing oxidative damage that impairs pathogenicity [43,44]. The present study revealed that AMB treatment significantly disrupted redox homeostasis in *B. cinerea*, with 49 oxidoreductase genes down-regulated and 78 up-regulated, ultimately attenuating virulence.

Upon attack from pathogens, plants initiate the production of phytoalexins, which are pivotal in strengthening their immune defense system [46]. However, bacteria and fungi possess the ability to detoxify plant toxins, thus enhancing their pathogenicity. Although targeted degradation of specific antimicrobial compounds might allow a pathogen to infect a particular host, generalized detoxification mechanisms, such as efflux, are highly valuable for pathogens capable of infecting diverse hosts [47]. Fungal pathogens employ ATP-binding cassette (ABC) and major facilitator superfamily (MFS) transporters as key detoxification systems, conferring resistance to diverse toxic compounds, both internally produced and externally sourced, including antibiotics, plant defense chemicals, and fungicides [48]. In *B. cinerea*, these transporters mediate both virulence and drug resistance through distinct mechanisms: (I) BcatrB mediates camalexin efflux, enhancing host colonization [49]; (II) the mitochondrial ABC transporter Bcmdl1 regulates spore germination and confers resistance to pyrazolopyrimidine fungicides [50]; and (III) MFS transporter mfsG provides protection against host-derived glucosinolate metabolites [48]. Parallel mechanisms exist in other fungal pathogens, as demonstrated by *Penicillium digitatum*, where the upregulation of *PdMFS2/3* significantly enhances tolerance to imazalil and thiabendazole fungicides [51]. Additionally, fungal pathogens utilize specialized transporter systems for host nutrient acquisition, a critical determinant of their reproductive success and virulence [51,52]. In this study, the expression of 28 genes related to transmembrane transport, including *Bcmfs1*, *Bchol1*, and *bcst1*, was down-regulated, indicating that AMB treatment might disrupt the efflux system of *B. cinerea* that detoxifies plant toxins, and affected nutrient acquisition, subsequently reducing the pathogenicity of *B. cinerea*.

Following that, we next examined the influence of AMB on the defense responses of table grapes. Previous studies have demonstrated that various antifungal compounds, including protocatechuic acid methyl ester [27], *p*-coumaric acid [28], melatonin [31], and cinnamaldehyde [53], confer disease resistance by boosting fruit antioxidant capacity and enhancing defense responses against fungal pathogens. This protective mechanism is mediated through the plant’s oxidative burst response, where pathogen recognition triggers rapid ROS accumulation that subsequently activates the antioxidant defense system to maintain cellular redox homeostasis [31]. In this study, the ROS-scavenging enzymes, such as CAT, SOD, and POD, showed elevated activities following AMB treatment. Moreover, pathogenesis-related proteins like PAL, PPO, CHI, and GLU are essential for the host to resist pathogens [27,54]. Notably, the comparative analysis demonstrated that these enzymatic activities were consistently higher in AMB-treated grapes than in untreated controls. Collectively, AMB treatment was found to enhance both antioxidant metabolic enzymes and defense-related protein activities in table grapes, resulting in effective suppression of *B. cinerea*-induced fruit decay.

This study has demonstrated that AMB effectively suppresses the growth of *B. cinerea* and mitigates the severity of gray mold in table grapes. Although cross-study comparisons present methodological challenges, AMB exhibits superior efficacy to alternative treatments, such as chitosan and essential oils [55,56,57,58], achieving greater fungal suppression at lower concentrations through its unique dual mode of action that combines direct pathogen inhibition with host defense potentiation. Unlike the fungistatic activity of chitosan or the volatility constraints of essential oils, AMB represents a distinctive postharvest disease control solution.

## 5. Conclusions

In summary, AMB exhibited strong antifungal activity against *B. cinerea*, with a minimal fungicidal concentration of 0.4 mg/L in vitro. Notably, at 200 mg/L, AMB completely inhibited gray mold development on table grapes, demonstrating its potential as an effective postharvest treatment. The underlying mechanism is attributed to the direct inhibition of mycelia growth and spore germination. Furthermore, AMB treatment disrupts the plasma membrane of *B. cinerea*, initiating cellular leakage and ultimately triggering cell death. The transcriptome analysis indicated that the antifungal effect of AMB also relies on modulating the function of peroxisomes, perturbing intracellular redox homeostasis, interfering with the efflux system, and impeding nutrient acquisition, ultimately leading to a reduction in the virulence of *B. cinerea*. In addition, AMB triggered the defense response of grapes to *B. cinerea* through the activation of ROS-scavenging enzymes and defensive enzymes, leading to a decrease in the occurrence of gray mold. Given the current findings, future research could explore the efficacy of AMB in additional perishable crops, the potential synergistic interactions between AMB and other antifungal agents, as well as the cost-effectiveness of commercial application of AMB.

## Data Availability

The original contributions presented in the study are included in the article/Supplementary Material, further inquiries can be directed to the corresponding author.

## Supplementary Materials

The following supporting information can be downloaded at https://www.mdpi.com/article/10.3390/foods14071260/s1: Table S1: Primers used in this article; Table S2: Differentially expressed genes between control and AMB-treated samples.
